# Association Between the Number of Chronic Diseases and Oral Health Problems in Korean Adults

**DOI:** 10.3290/j.ohpd.b4925339

**Published:** 2024-02-01

**Authors:** Hee Jin Lee, Youn Huh, Sung Sunwoo

**Affiliations:** a Doctor, International Healthcare Center, Asan Medical Center, Seoul, Republic of Korea. Wrote the original draft, contributed to the statistical analysis, discussed the results, accepted equal responsibility for the accuracy and content of the paper, read and approved the final manuscript.*; b Professor, Department of Family Medicine, Uijeongbu Eulji Medical Center, Eulji University, Gyeonggi-do, Republic of Korea. Wrote the original draft manuscript, contributed to the statistical analysis, discussed the results, accepted equal responsibility for the accuracy and content of the paper, read and approved the final manuscript.*; c Professor, Department of Family Medicine, Asan Medical Center, University of Ulsan College of Medicine, Seoul, Republic of Korea. Revised the manuscript and provided supervision, accepted equal responsibility for the accuracy and content of the paper, read and approved the final manuscript.; * Contributed equally to the manuscript.

**Keywords:** chronic diseases, dental treatment, Korea, multimorbidity, oral health problems

## Abstract

**Purpose:**

The relationship between the number of chronic diseases and oral health problems is unclear. We sought to determine whether the number of chronic diseases and multimorbidity have an association with oral health problems in Korean adults.

**Materials and Methods:**

Data from 23,246 adults aged ≥ 19 years, who participated in the Korea National Health and Nutrition Examination Survey from 2016 to 2019, were considered for our analyses. Participants with either masticatory or speech problems were defined as the oral health problems group. Individuals who reported having had dental treatment in the last year were defined as the dental treatment group. We used multivariable logistic regression analyses to calculate odds ratios (ORs) and 95% confidence intervals (CIs).

**Results:**

The proportions of oral health problems and dental treatment were higher in participants with multimorbidity than in those without multimorbidity (all p < 0.001). Moreover, ORs of oral health problems demonstrated a tendency to increase with the number of chronic diseases, even after adjustment (p for trend < 0.001). Compared to the participants without multimorbidity, the risk of having oral health problems increased by 25% (OR: 1.25, 95% CI: 1.12–1.39), and that of receiving dental treatment increased by 23% (OR: 1.23, 95% CI: 1.13–1.34) in patients with multimorbidity.

**Conclusion:**

The risk of oral health problems and dental treatment increased in association with the number of chronic diseases in Korean adults. The authors emphasise the risks and importance of oral health in a large population affected by multiple chronic diseases.

Chronic diseases are the main cause of mortality in most adults.^17^ People with chronic diseases have a decreased quality of life, increased health care costs, and a high risk of mortality.^1,15^ Moreover, the prevalence of chronic diseases is rapidly rising in aging societies. The occurrence of multiple health conditions is referred to as multimorbidity and is commonly defined as the coexistence of two or more diseases.^9^ In studies conducted with 10 chronic diseases, including cancer, lung disease, heart disease, asthma, diabetes mellitus, hypertension, stroke, and kidney disease, the prevalence of ≥ 2 diseases in US adults aged ≥ 18 years was 22% in 2001 and 26% in 2012.^24,25^ Approximately 52% of US adults had one or more of the 10 chronic diseases; of these, among adults aged ≥ 18 years in 2018, 27% had ≥ 2 diseases.^3^

Chronic diseases and dental health are inseparable conditions that share common risk factors, such as poor diet and tobacco use.^21^ Furthermore, a bidirectional relationship has been noted in existing literature.^7,11^ Associations between dental and medical conditions have received much attention, and ranging from cardiometabolic diseases to inflammatory bowel disease, kidney disease, rheumatic arthritis, and even Alzheimer’s disease.^7,10,12,13,18,19^ Periodontal microorganisms or their cellular components – resulting in local inflammation and their consequent translocation leading to systemic inflammation – are known to serve as a link to various diseases.^19^ In addition to disease-specific aetiologies that contribute to dental diseases, age-specific issues are also present in older populations. In older individuals, multiple prescribed medications for multimorbidity induce xerostomia, which may increase the risk of dental diseases.^6,10^ Patients with severe chronic diseases often suffer from masticatory and eating problems due to dental diseases. This may be a factor contributing to a reduced quality of life.^8,20^ However, studies that examined the relationship between the number of chronic diseases and multimorbidity with oral health problems among adults are limited.

Therefore, we sought to determine the relationship between the number of chronic diseases or multimorbidity with oral health problems. Additionally, we aimed to analyse the relationship between the number of chronic diseases and multimorbidity with oral health problems among people aged ≥ 19 years, using the Korea National Health and Nutrition Examination Survey (KNHANES). We also performed stratified analyses depending on age, sex, and body mass index (BMI).

## Materials and Methods

### Data Source and Study Participants

Data from participants who completed the KNHANES between 2016 and 2019 were used. The KNHANES is a nationwide survey that measures the state of health and nutrition in South Korea. The aforementioned surveys are designed to identify vulnerable populations that require prioritisation and to evaluate the effectiveness of present health policies. The KNHANES consists of a nutrition survey, a health examination, and a health interview. Information from personal interviews and data from physical examinations as well as blood and urine samples were collected at a mobile examination facility.

Our study initially selected 37,753 individuals who participated in KNHANES between 2016 and 2019. From this group, individuals aged ≤ 18 years (n = 9376) and those missing key variables (n = 5131) were excluded. Finally, 23,246 participants aged ≥ 19 years were included in our analyses. As the questionnaire items about the presence or absence of chronic diseases in KNHANES were targeted for participants aged ≥ 19, our study participants were also aged ≥ 19. This study adhered to the principles of the Declaration of Helsinki, and written informed consent was obtained from all adults participating in our study.

### Definition of Chronic Diseases

The KNHANES provided the list of chronic diseases, which included cardiovascular diseases (hypertension, dyslipidemia, myocardial infarction, stroke), endocrine diseases (diabetes mellitus, thyroid disease), liver diseases (liver cirrhosis, hepatitis), allergic diseases (atopic dermatitis, asthma), arthritis, renal failure, and cancer (stomach cancer, colon cancer, breast cancer, liver cancer, lung cancer, thyroid cancer, cervical cancer, and others). Finally, the chronic disease group was defined as those who responded to having been diagnosed by a physician with one of the above chronic diseases.

### Definition of Oral Health Problems and the Dental Treatment Group

In the oral health surveys, the following question was asked: “Do you currently have problems in your mouth, such as those involving teeth, dentures, or gums, making chewing food difficult?” Participants who responded with “moderate”, “uncomfortable”, or “very uncomfortable” were defined as the masticatory-problems group. Also, participants were asked the following: “Are you experiencing difficulty or discomfort in pronouncing clearly due to problems in your mouth, such as those involving teeth, dentures, or gums?” Participants who responded with “moderate”, “uncomfortable”, or “very uncomfortable” were defined as the speech-problems group. Participants with at least one masticatory or speech problem were defined as having oral health problems. Another question asked in the oral health survey was: “Have you had dental treatment in the last year?” Participants who responded “yes” and received treatment for gum disease, caries, or dental nerve involvement were defined as the dental-treatment group.

### Other Covariates

Household income was classified into four categories. Participants were divided into two groups based on whether they had more than or equal to 9 years of schooling (middle school graduation). Current smokers were defined as those who had smoked more than 100 cigarettes in their lives and were still smoking at the time of the study. Men who consumed more than or equal to two drinks per day and women who consumed more than or equal to one drink per day were referred to as heavy alcohol drinkers.^2^ Regular exercise was categorised as exercising more than or equal to three times per week. Moreover, BMI was calculated by dividing the weight in kg by height in m^2^.

### Statistical Analysis

We combined the raw data from the KNHANES between 2016 and 2019 according to the raw data-analysis guidelines. Furthermore, since the data were derived from a complex sample design, all analyses were conducted according to the weighted sample values and the assigned clustering and stratification variables. A chi-squared test was performed to evaluate the proportions of dental treatment and oral health problems, according to chronic disease morbidity and the number of chronic diseases. Consequently, a multivariable logistic regression analysis was conducted to calculate the odds ratio (OR) and 95% confidence intervals (CIs) to assess the association between chronic disease morbidity with dental treatment and oral health problems. The analysis was adjusted for age, sex, household income, educational level, alcohol consumption, smoking status, and physical activity. Moreover, we also performed stratified analyses depending on age, sex, and BMI. A p-value < 0.05 was considered statistically significant. SPSS Statistics 24.0 (IBM; Armonk, NY, USA) was used for analyses.

## Results

### Characteristics of the Study Participants

Table 1 summarises the basic characteristics of participants. Data from 23,246 adults, consisting of 10,173 (49.6%) men and 13,073 (50.4%) women, were used for analyses. The proportion of lower income and educational level was higher in the oral health problems group than in the group without oral health problems (p < 0.001). The proportion of current smokers was also higher in participants with oral health problems (p < 0.001). However, the proportion of risky drinkers and individuals who exercised regularly was higher in the group without oral health problems (p < 0.001). The proportion of people who received dental treatment was higher in the group with oral health problems than in the healthy group (p < 0.001).

**Table 1:** Basic characteristics of participants

	Oral problems (+)		Oral problems (-)		Total		p-value
	n = 3597		n = 19,649		n = 23,246		
**Age (years)**							
19–39	335	14.6 (0.9)	6132	39.1 (0.6)	6467	35.8 (0.6)	< 0.001
40–64	1429	47.5 (1.1)	9464	47.9 (0.5)	10,893	47.8 (0.5)	
≥65	1833	37.9 (1.1)	4053	13.1 (0.4)	5886	16.3 (0.4)	
**Sex**							
Male	1631	49.0 (1.0)	8542	49.7 (0.4)	10,173	49.6 (0.3)	0.53
Female	1966	51.0 (1.0)	11,107	50.3 (0.4)	13,073	50.4 (0.3)	
**Education (years)**							
≤9	2128	50.0 (1.2)	4804	18.2 (0.5)	6932	22.4 (0.5)	< 0.001
**Income**							
Lowest	1406	33.3 (1.1)	2988	12.6 (0.4)	4394	15.3 (0.5)	< 0.001
**Cigarette smoking**							
Non-smoker	1986	51.6 (1.0)	12,459	60.4 (0.4)	14,445	59.2 (0.4)	< 0.001
Ex-smoker	846	22.4 (0.8)	3804	19.3 (0.3)	4650	19.7 (0.3)	
Current smoker	765	26.0 (1.0)	3386	20.4 (0.4)	4151	21.1 (0.4)	
**Alcohol consumption**							
Non-risky drinker	1952	49.0 (1.1)	8703	40.1 (0.5)	10,655	41.3 (0.4)	< 0.001
Risky drinker	1645	51.0 (1.1)	10,946	59.9 (0.5)	12,591	58.7 (0.4)	
**Physical activity**							
Irregular	2413	64.3 (1.1)	10,783	52.0 (0.5)	13,196	53.6 (0.5)	< 0.001
Regular	1184	35.7 (1.1)	8866	48.0 (0.5)	10,050	46.4 (0.5)	
**BMI (kg/m^2^)**							
<18.5	133	3.7 (0.4)	749	4.1 (0.2)	882	4.0 (0.2)	0.13
18.5–24.9	2122	59.6 (1.0)	12,176	61.3 (0.4)	14,298	61.1 (0.4)	
≥25	1342	36.7 (1.0)	6724	34.6 (0.4)	8066	34.9 (0.4)	
**Dental treatment**							
(+)	1228	35.3 (1.0)	5664	28.2 (0.4)	6892	29.2 (0.4)	< 0.001

BMI: body mass index. *Results are calculated from complex sample analysis and standardised by direct method. Results are expressed as unweighted number and weighted proportion (standard error).

### Proportions of Oral Health Problems and Dental Treatment Depending on the Number of Chronic Diseases and Multimorbidity

52.6% of the study participants had no chronic diseases. Oral health problems and dental treatment were reported in 15.5% and 29.6% of the participants, respectively. Table 2 exhibits the prevalence of oral health problems and dental treatment depending on the number of chronic diseases and multimorbidity. Oral health problems were present in 8.6%, 15.7%, 20.3%, 26.0%, and 29.1% of the participants with 0, 1, 2, 3, and ≥ 4 chronic diseases, respectively (p for trend < 0.001). A history of dental treatments was present in 27.2%, 30.8%, 32.8%, 33.0%, and 34.4% of the participants with 0, 1, 2, 3, and ≥ 4 chronic diseases, respectively (p for trend < 0.001). The proportions of oral health problems and dental treatment were higher in participants with multimorbidity than those without multimorbidity (all p < 0.001).

**Table 2:** Prevalence of dental treatment and oral problems according to the number of chronic diseases and multimorbidity

	Total	Dental treatment (+) (N = 6892)			Oral problems (+) (N = 3597)		
**Number of chronic diseases**				**p for trend**			**p for trend**
0	12,223	3409	27.2 (0.5)	< 0.001	1154	8.6 (0.3)	< 0.001
1	4871	1493	30.8 (0.8)		881	15.7 (0.6)	
2	3242	1039	32.8 (1.0)		726	20.3 (0.9)	
3	1796	581	33.0 (1.3)		496	26.0 (1.2)	
≥4	1114	370	34.4 (1.7)		340	29.1 (1.6)	
**Multimorbidity**				**p-value**			**p-value**
(+)	6152	3483	32.0 (0.5)	< 0.001	2443	19.7 (0.5)	< 0.001
(-)	17,094	3409	27.2 (0.5)		1154	8.6 (0.3)	

* Results are calculated from complex sample analysis and standardised by direct method. Results are expressed as unweighted number and weighted proportion (standard error). Values were obtained by using chi-squared tests.

### Associations Between the Number of Chronic Diseases and Multimorbidity with Oral Health Problems

Table 3 displays the results of multivariable logistic regression analysis regarding the association between the number of chronic diseases and multimorbidity with oral health problems. The prevalence of oral health problems demonstrated a concurrent tendency to increase with multimorbidity (p for trend < 0.001). Moreover, in all the adjusted models, the ORs of having oral health problems tended to increase as the number of chronic diseases increased (p for trend < 0.001). In individuals with four or more comorbid chronic diseases, the risk of having oral health problems was 1.54 times higher (OR: 1.54, 95% CI: 1.27–1.87) compared to those with no chronic diseases. Even when stratified according to age, sex, and BMI, the ORs of dental treatment increased with the increasing number of chronic diseases, except for those aged ≥ 65 years. In terms of a multimorbid state, the risk of oral health problems increased by 25% (95% CI: 1.12–1.39) in the multimorbid group compared to that in the control group.

**Table 3:** Associations between the number of chronic diseases or multimorbidity and oral health problems

		Number of diseases					p for trend	Multimorbidity	
		0	1	2	3	≥4		(-)	(+)
		aOR (95% CI)						aOR (95% CI)	
Total		1	1.23 (1.09–1.39)	1.30 (1.13–1.50)	1.45 (1.22–1.71)	1.54 (1.27–1.87)	< 0.001	1	1.25 (1.12–1.39)
Age (years)	40–64	1	1.34 (1.14–1.58)	1.39 (1.14–1.70)	1.62 (1.25–2.12)	1.91 (1.37–2.65)	< 0.001	1	1.36 (1.17–1.58)
	≥65	1	1.10 (0.90–1.35)	1.11 (0.90–1.37)	1.17 (0.93–1.48)	1.23 (0.97–1.56)	0.09	1	1.08 (0.95–1.24)
Sex	Male	1	1.15 (0.96–1.38)	1.27 (1.04–1.57)	1.63 (1.27–2.09)	1.76 (1.31–2.37)	< 0.001	1	1.35 (1.16–1.57)
	Female	1	1.31 (1.10–1.55)	1.30 (1.07–1.57)	1.31 (1.04–1.64)	1.40 (1.10–1.80)	0.004	1	1.15 (1.00–1.34)
BMI (kg/m^2^)	<25	1	1.23 (1.06–1.44)	1.34 (1.11–1.60)	1.35 (1.09–1.68)	1.51 (1.14–1.99)	< 0.001	1	1.24 (1.08–1.43)
	≥25	1	1.24 (1.00–1.54)	1.24 (0.99–1.54)	1.55 (1.19–2.01)	1.57 (1.19–2.07)	0.006	1	1.25 (1.06–1.47)

aOR: adjusted odds ratio; CI: confidence interval; BMI: body mass index. Model is adjusted for age, sex, household income, education, cigarette smoking, alcohol drinking, and physical activity. Values were calculated by multivariable logistic regression analysis.

### Associations Between the Number of Chronic Diseases and Multimorbidity with Dental Treatment

Table 4 displays the data from the multivariable logistic regression analysis regarding the relationship between the number of chronic diseases and multimorbidity with dental treatment. Even after adjustment, the OR for receiving dental treatment increased with the number of chronic conditions (p for trend < 0.001). When an individual had four or more chronic diseases, the OR of having dental treatment was 1.45 (95% CI: 1.23–1.72) compared to those with no chronic comorbidities. Even when stratified according to age, sex, and BMI, the ORs of dental treatment increased with the number of chronic conditions, except for those aged 19–39 years. Compared to the participants without multimorbidity, the risk of receiving dental treatment increased by 23% (OR: 1.23, 95% CI: 1.13–1.34) in those with multimorbidity.

**Table 4:** Associations between the number of chronic diseases or multimorbidity and dental treatment

		Number of diseases					p for trend	Multimorbidity	
		0	1	2	3	≥4		(-)	(+)
		aOR (95% CI)						aOR (95% CI)	
Total		1	1.16 (1.06–1.26)	1.26 (1.13–1.41)	1.33 (1.16–1.52)	1.45 (1.23–1.72)	< 0.001	1	1.23 (1.13–1.34)
Age (years)	19–39	1	1.07 (0.88–1.28)	1.28 (0.86–1.90)	1.65 (0.71–3.83)	2.58 (0.46–14.44)	0.07	1	1.35 (0.94–1.93)
	40–64	1	1.23 (1.10–1.36)	1.31 (1.15–1.49)	1.34 (1.12–1.61)	1.36 (1.04–1.78)	< 0.001	1	1.24 (1.12–1.38)
	≥65	1	0.99 (0.80–1.22)	1.07 (0.86–1.33)	1.15 (0.93–1.43)	1.35 (1.07–1.71)	0.005	1	1.16 (1.02–1.33)
Sex	Male	1	1.17 (1.04–1.33)	1.24 (1.06–1.46)	1.24 (1.01–1.53)	1.58 (1.20–2.08)	< 0.001	1	1.21 (1.06–1.37)
	Female	1	1.15 (1.02–1.29)	1.28 (1.10–1.50)	1.39 (1.16–1.67)	1.41 (1.14–1.74)	< 0.001	1	1.26 (1.12–1.42)
BMI (kg/m^2^)	<25	1	1.15 (1.03–1.29)	1.29 (1.12–1.49)	1.40 (1.17–1.68)	1.64 (1.31–2.06)	< 0.001	1	1.29 (1.16–1.44)
	≥25	1	1.16 (1.01–1.35)	1.21 (1.02–1.45)	1.24 (1.01–1.52)	1.30 (1.02–1.65)	< 0.001	1	1.16 (1.01–1.33)

aOR: adjusted odds ratio; CI: confidence interval; BMI: body mass index. Model is adjusted for age, sex, household income, education, cigarette smoking, alcohol drinking, and physical activity. Values were calculated by multivariable logistic regression analysis.

## Discussion

The data supporting the authors’ conclusions in this study are publicly available at the Korea Centers for Disease Control and Prevention. To request the data, please visit the following link: https://knhanes.cdc.go.kr/knhanes/main.do

In this study, we investigated the association between the number of chronic diseases and oral health problems in Korean adults. The proportion of people receiving dental treatment and the prevalence of oral health problems was higher in participants with multimorbidity than those without it. The risk of having oral health problems increased with the number of chronic diseases, especially when an individual was comorbid with ≥ 4 chronic diseases. The OR was 1.54 compared to those with no chronic diseases. This trend was still present when participants were stratified according to age, sex and BMI. Our results imply that the state of multimorbidity is associated with poor oral health.

The association between chronic disease and dental status has been studied in the existing literature. However, since they are entities dealt with by two different sectors, dental and medical, a comprehensive understanding of their relation and close communication is required for integrative health care. Even though the mechanisms by which an individual chronic disease affects dental health have been studied, the number of chronic comorbidities as a factor for poor oral health has not been assessed.

To add to the current knowledge of the dynamics between chronic diseases and oral health, this study sought to determine whether a high number of chronic diseases is a risk factor for oral health problems and dental treatments.

Previous studies have demonstrated associations between dental conditions and specific chronic diseases. The most frequently studied correlation between dental disease and chronic disease was that of periodontitis with type-2 diabetes mellitus and cardiovascular diseases.^22^ Also, in a Korean cohort study where the number of teeth in four chronic diseases (diabetes mellitus, hypertension, osteoporosis, and rheumatoid arthritis) was analysed, the tooth loss per person was high in the diabetes mellitus, hypertension, and rheumatoid arthritis groups.^26^

The mechanisms by which chronic disease causes dental health issues have also been studied. In the case of diabetes mellitus, patients have more dental plaque, which increases the future risk of caries and tooth loss.^4^ Regarding diabetic neuropathy, glucotoxicity is the primary risk factor that induces downstream metabolic and microvascular effects that cause oxidative stress, chronic inflammation, and peripheral nerve fiber damage.^4^ Also, patients with type-2 diabetes mellitus display greater xerostomia than do controls,^5^ which is known to increase the risk of dental diseases among older people.^6^ More recent evidence demonstrates that other factors such as age, hypertension, hyperlipidemia, and obesity also play important roles as common mechanisms for diabetic neuropathy and periodontitis.^4^

Our study demonstrated that the risk of oral problems and dental treatment increased according to the number of chronic diseases and the state of multimorbidity in Korean adults. The same trend was observed even in subgroup analyses. The high number of chronic diseases increases the risk of oral health problems independent of age, sex, and BMI, which are well-known common risk factors of poor oral health.^22^ Even when stratified according to age, sex, and BMI, the risk of oral health problems and dental treatment still increased in the multimorbid group. Regardless of the BMI, the risk of oral health problems increased with the number of chronic diseases, and the OR was not high in the group with obesity (BMI ≥ 25). In addition to the results observed in a population study based on Korean adults where the risk of tooth extraction was highest in the age group over 70 years in both sexes,^26^ our results add to the current knowledge that a high number of chronic diseases increases the risk of dental treatment independent of age, which is a well-known risk factor for dental problems.^22^

The finding that a multimorbid state was associated with more dental problems that require consequent treatments heightens emphasis on future inter-sectoral patient care in the medical and dental fields. An intimate collaboration with dental specialists is required for physicians considering the high prevalence of the multimorbid population. Furthermore, in an aging society, preserving the number of teeth is directly related to maintaining good oral health and is consequently important to improve the quality of life and life expectancy in the older population.^14^ The present study demonstrated an association similar to that displayed by the previous study;^14^ however, while the previous study was limited to older people, our study included all adults.

This study has some limitations. First, as this was a cross-sectional study, causal relationships between multimorbidity and dental problems could not be identified. Second, since the KHANES data is based on self-reports of the participant’s disease status, the data may be subject to bias. Lastly, other than the several factors that we have taken into consideration that may influence oral problems, this study might not have fully accounted for other existing confounding variables that contribute to either medical or dental conditions, as well as factors that contribute as causative risks for both diseases. However, our study revealed the important association between multimorbidity and dental problems among Korean adults.

## Conclusion

Our study provides a generalised insight into the relationship between “multimorbid status” and dental health according to the number of chronic diseases. By defining “chronic disease” as including a wide range of medical conditions, this study emphasised the risks and importance of oral health in a large population affected by multiple chronic diseases.
